# Reconfigurations in brain networks upon awakening from slow wave sleep: Interventions and implications in neural communication

**DOI:** 10.1162/netn_a_00272

**Published:** 2023-01-01

**Authors:** Cassie J. Hilditch, Kanika Bansal, Ravi Chachad, Lily R. Wong, Nicholas G. Bathurst, Nathan H. Feick, Amanda Santamaria, Nita L. Shattuck, Javier O. Garcia, Erin E. Flynn-Evans

**Affiliations:** Fatigue Countermeasures Laboratory, Department of Psychology, San José State University, San José, CA, USA; Department of Biomedical Engineering, Columbia University, New York, NY, USA; US DEVCOM Army Research Laboratory, Humans in Complex Systems Division, Aberdeen Proving Ground, MD, USA; Fatigue Countermeasures Laboratory, Human Systems Integration Division, NASA Ames Research Center, Moffett Field, CA, USA; Cognitive and Systems Neuroscience Research Hub, University of South Australia, Adelaide, SA, Australia; Operations Research Department, Naval Postgraduate School, Monterey, CA, USA

**Keywords:** Sleep inertia, Short-wavelength-enriched light, Network communication, Graph theoretical framework

## Abstract

Sleep inertia is the brief period of impaired alertness and performance experienced immediately after waking. Little is known about the neural mechanisms underlying this phenomenon. A better understanding of the neural processes during sleep inertia may offer insight into the awakening process. We observed brain activity every 15 min for 1 hr following abrupt awakening from slow wave sleep during the biological night. Using 32-channel electroencephalography, a network science approach, and a within-subject design, we evaluated power, clustering coefficient, and path length across frequency bands under both a control and a polychromatic short-wavelength-enriched light intervention condition. We found that under control conditions, the awakening brain is typified by an immediate reduction in global theta, alpha, and beta power. Simultaneously, we observed a decrease in the clustering coefficient and an increase in path length within the delta band. Exposure to light immediately after awakening ameliorated changes in clustering. Our results suggest that long-range network communication within the brain is crucial to the awakening process and that the brain may prioritize these long-range connections during this transitional state. Our study highlights a novel neurophysiological signature of the awakening brain and provides a potential mechanism by which light improves performance after waking.

## Author Summary

Using a graphical framework approach, our findings suggest that following awakening from slow wave sleep: (a) a prioritization scheme may underlie recovery rates for different behaviors; (b) long-range neural connections orchestrating local-global operations are uniquely disrupted; and (c) light is able to minimize disruption to long-range connections, revealing the potential mechanism through which light acts as a countermeasure. This research (a) advances the knowledge of neural processes during the transition from sleep to wakefulness; (b) demonstrates a mechanism underlying a brain-behavior relationship that may serve as a target for future countermeasure research; and (c) applies a novel methodological approach to sleep-wake brain states. Further research is needed to apply this analytical method to alternative interventions and sleep-wake transition scenarios.

## INTRODUCTION

Immediately after waking from sleep there is a temporary period of reduced alertness and performance. The impact of this so-called sleep inertia on behavioral performance measurements has been well described, including impaired reaction times (Hilditch et al., 2016; Van Dongen et al., 2001), memory (Achermann et al., 1995; Santhi et al., 2013), decision-making (Bruck & Pisani, 1999; Horne & Moseley, 2011), and a variety of other cognitive functions (Burke et al., 2015). These behaviors are also associated with a perceived state of sleepiness (Burke et al., 2015; Santhi et al., 2013), disorientation (Dinges, 1990), poor mood (Hilditch et al., 2022), and misperceptions of performance (Hilditch et al., 2016).

Existing sleep inertia research has associated the waking process with several neural changes that include increased delta power over posterior regions of the brain (Ferrara et al., 2006; Marzano et al., 2011), reduced beta power across all brain regions (Marzano et al., 2011), and increased functional connectivity of the default mode network (Vallat et al., 2019). Interestingly, the links between observed impaired performance and the neural behavior during sleep inertia are also commonly associated with sleep-related neural processes and states of sleepiness due to homeostatic and circadian pressures (Aeschbach et al., 1997). These observations suggest a complex orchestration of neural elements supporting the transition from sleep to wakefulness spanning these oppositional constructs. Preliminary research investigating this complexity in neural network changes has suggested broad functional connectivity changes post-sleep, with the default mode network and the delta and beta bands playing a critical role in the network changes transitioning from sleep to wakefulness (Chen et al., 2020; Vallat et al., 2019). These connectivity changes, however, have yet to be characterized and it is unknown whether an intervention may moderate these brain changes.

Understanding how heterogeneous neural elements of the brain coalesce to produce behavior and subjective experience may be understood via a graph theoretical framework. Using this framework, the brain is visualized as a graph or network made up of a collection of nodes (specified brain regions) and edges (connections) that represent brain elements and the corresponding statistical relationship between them. Topological description of brain networks within this framework can provide quantitative insights into the underlying mechanisms that give rise to emerging neural properties such as specialization and efficiency of information processing (Bassett & Sporns, 2017), a variety of cognitive phenomena (Bassett & Sporns, 2017; Garcia et al., 2018; Medaglia et al., 2015), transitioning brain states (Miraglia et al., 2021), and abnormalities in neurological disorders (Y. Liu et al., 2008). Two common metrics, clustering coefficient and path length, have been used to describe properties of many complex systems, from biological phenomena (Watts & Strogatz, 1998) to higher level systems (Telesford et al., 2011). Clustering coefficient estimates the tendency of a node’s neighbors within a network to also be linked. Path length, on the other hand, estimates the number of edges, on average, that must be traversed to connect any two nodes within a network. When these metrics are at intermediate levels, associated with neither random nor regular networks, they describe the properties of a small-world network, which has been established as a popular model to describe functional brain networks by facilitating both localized and distributed processing of information.

In the current study, we describe the neurophysiological profile of the awakening brain under ecologically relevant sleep and circadian pressures using graph theoretical analysis of functional connectivity with 32-channel electroencephalography (EEG). Using clustering and path length, we see that sleep inertia is characterized by a global shift in these metrics immediately after awakening. Moreover, we confirm, using a within-subject, randomized, crossover intervention design, that polychromatic short-wavelength-enriched light exposure alleviates this sleep inertia effect. We interpret our findings within the context of discontinuity of neural elements and efficiency of brain processes while the brain transitions from sleep to wakefulness.

## RESULTS

**Figure 1.**
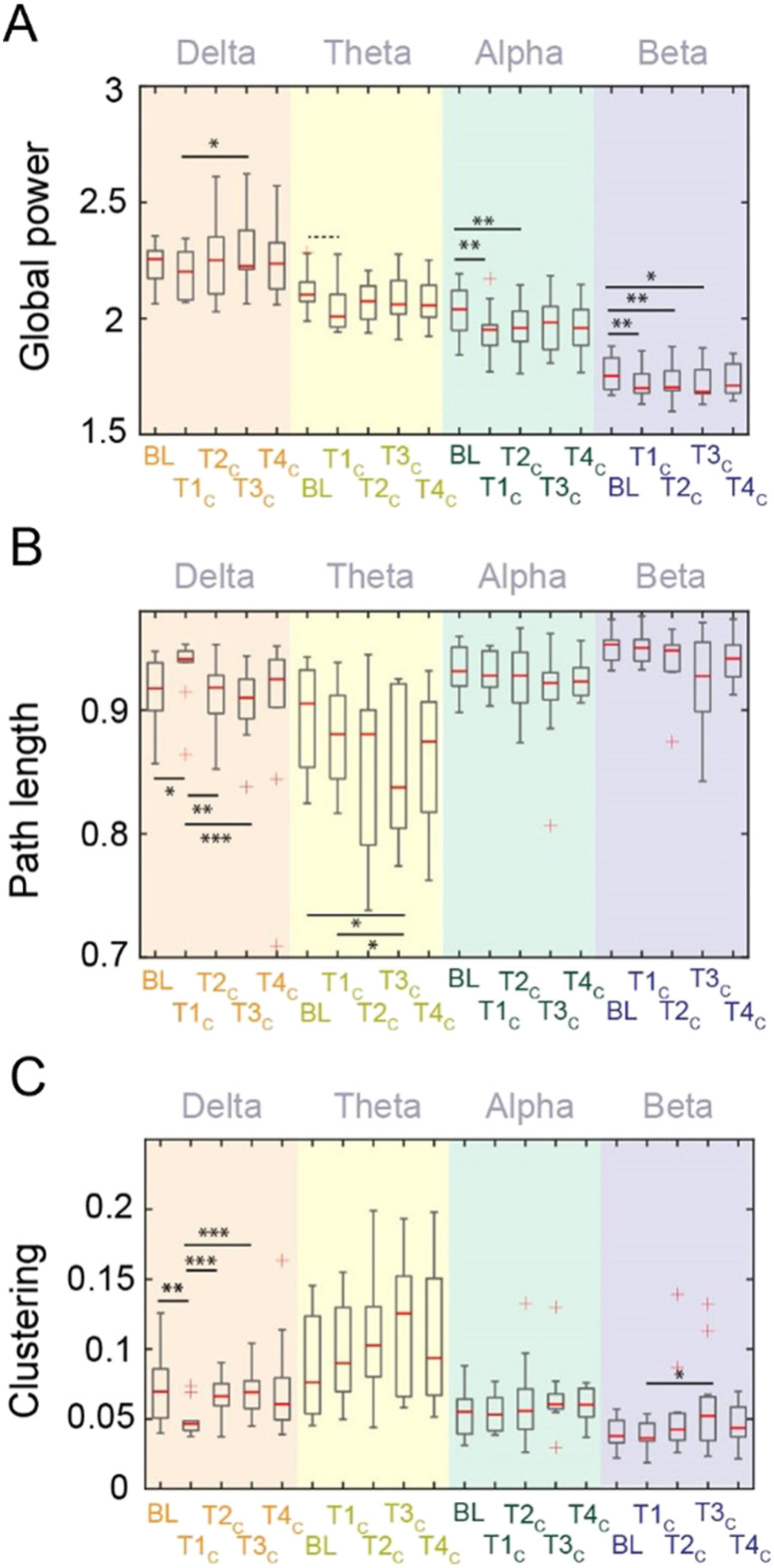
Comparison for (A) power and (B), (C) brain network properties across test bouts for each frequency band in the control condition (dim red light). BL = baseline, T#_C_ = Test bout # during the control condition. Asterisks represent significant difference on a paired *t* test without any further correction applied such that *p < 0.05, **p < 0.01, ***p < 0.001. Dashed line denotes marginally significant difference (*p* = 0.053).

EEG was analyzed from 11 participants (6 female; 23.1 ± 4.4 years, range 19–35 years). Data were recorded while participants performed a reaction time task (psychomotor vigilance task, PVT) following nocturnal awakenings from slow wave sleep (SWS). Table S1 in the Supporting Information contains sleep history information; see Hilditch et al. (2022) for details of sleep macrostructure prior to awakenings. Briefly, because of the functional associations with defined categories of oscillations within the brain (Nunez & Srinivasan, 2006) and previous associations of band-specific spectral power and sleep inertia (Ferrara et al., 2006; Marzano et al., 2011; Vallat et al., 2019), we first assessed the evolution of *global* spectral power (i.e., average across all channels) of the EEG recorded during the four separate test bouts and compared them with the pre-sleep baseline assessment. Then, via pairwise connectivity (wPLI) to estimate the phase-based relationship between channels, we estimated graph metrics that purposefully targeted the segregating and integrating aspects of the network. The temporal evolution of these estimates were then assessed to evaluate the duration of sleep inertia. Previous studies assessing the impact of sleep inertia typically report severe behavioral impairments that resolve within 15–30 min of waking (Hilditch & McHill, 2019). Remaining mild impairment may take up to an hour or more to dissipate, depending on conditions such as sleep pressure, sleep stage at awakening, and time of day (Hilditch & McHill, 2019; Jewett et al., 1999). Given these previous findings and our behavioral results under similar conditions (Hilditch et al., 2016; Hilditch et al., 2022), we consider a neural change to be due to sleep inertia when, compared with baseline, there is a significant change in the assessed metric at the first test bout (i.e., 2 min after awakening; Figure 1, BL vs. T1_C_).

### Global Power of Lower Frequencies Recovers Faster Than Higher Frequencies During Sleep Inertia

Figure 1A displays the average spectral power across all channels as a function of test bout. Statistical comparisons between these test bouts within each frequency band describe a complex coordination of neural firing that may be initially disturbed upon awakening but then gradually recovers. Compared with participants’ global beta power prior to sleep (following moderate sleep restriction), beta global power was significantly reduced at T1_C_ (*t*(10) = 3.47, *p* = 0.006, effect size Hedge’s *g* = 0.6), T2_C_ (*t*(10) = 3.54, *p* = .005, *g* = 0.56), and T3_C_ (*t*(10) = 2.98, *p* = 0.014, *g* = 0.47). Similarly, compared with baseline, global alpha power was significantly reduced in the first two test bouts in the control (dim red light) condition (T1_C_: *t*(10) = 3.84, *p* = 0.003, *g* = 0.76; T2_C_: *t*(10) = 3.56, *p* = 0.005, *g* = 0.66). In the theta band, compared with baseline, we observed that the global power was marginally lower only at T1_C_ (*t*(10) = 2.2, *p* = 0.053, *g* = 0.71). There were no significant differences between baseline and post-awakening test bouts within the delta band (all *p* > 0.05). Similar patterns of global power were observed under equivalent testing conditions performed one week apart (see Figure S1A in the Supporting Information). To better understand the specificity of these findings, we also explored aperiodic components effects (Donoghue et al., 2020) with the preprocessing steps used here (Figure S2A in the Supporting Information) and those specific to the aperiodicity analysis (Figure S2C in the Supporting Information). We find that the aperiodic components using the preprocessing steps here displayed sustained differences between the pre-sleep baseline and subsequent temporal intervals after awakening, most similar to the findings within the higher frequencies of global power (see Figure S2 in the Supporting Information). Overall, though, our results presented here are not driven by aperiodic signals in the EEG; however, using another preprocessing pipeline did show promise for this analysis. For an extended discussion on the effects of preprocessing on these aperiodic results, see the Supporting Information.

### Network Properties of Delta Band Connectivity Display Unique Characteristics During Sleep Inertia

Next, we considered the global graph metrics of average shortest path length as a measure of integration and communication efficiency, in addition to average clustering coefficient as a metric of segregation. Under control conditions, path length was significantly increased in the delta band immediately after awakening compared with the pre-sleep baseline (*t*(10) = −2.52, *p* = 0.03, *g* = 0.73). Path length reached baseline levels at T2_C_ (Figure 1B). Similarly, we observed a significant reduction of clustering coefficient immediately after awakening from SWS at night (T1_C_) compared with pre-sleep baseline (*t*(10) = 4.0, *p* = 0.002, *g* = 1.17; Figure 1C). The clustering coefficient also returned to baseline levels at T2_C_ and persisted at that level. Except for a single effect of path length in the theta band significantly decreasing more than 30 min after awakening (*t*(10) = 2.65, *p* = 0.02, *g* = 0.76), there were no significant differences in these metrics compared with baseline in other frequency bands. Thus, path length and clustering coefficient within the delta band reflect a robust sleep inertia signal, with an initial significant change immediately after awakening, followed by recovery towards baseline at later time points. Similar patterns in delta band connectivity were observed under equivalent testing conditions performed one week apart (see Figure S1B–C in the Supporting Information).

### Polychromatic Short-Wavelength-Enriched Light Exposure at Awakening Attenuates Neural Network Changes Associated With Sleep Inertia in the Delta Band

**Figure 2.**
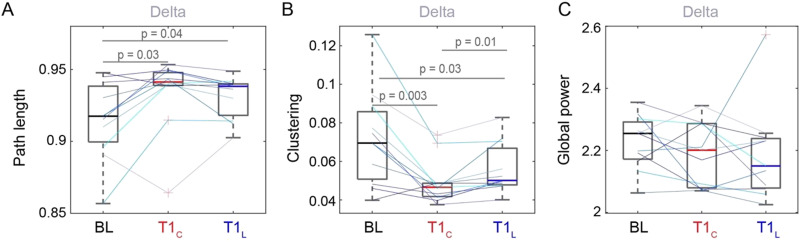
Brain network properties comparing pre-sleep baseline (BL), control at T1 (T1_C_), and light at T1 (T1_L_) for (A), (B) the delta frequency band and (C) delta power. Colored lines represent individual participants. Here, *p* represents the *p* value on a paired *t* test without any further correction applied.

We also assessed the effect of polychromatic short-wavelength-enriched light as a way to potentially alleviate the neural changes associated with sleep inertia. Indeed, previous studies have shown that short-wavelength-enriched light has acute alerting properties, especially at night (Lok et al., 2018; Souman et al., 2018), and this effect has been demonstrated during the sleep inertia period (Hilditch et al., 2022). Figure 2 displays the effect of polychromatic short-wavelength-enriched light on sleep inertia for each of the estimated global metrics across the frequency bands of interest for the baseline condition, the control condition at T1 (T1_C_), and the light intervention condition also at T1 (T1_L_). Importantly, we were interested in the effects that showed a difference between the control condition and the light intervention condition at this first time point at which the sleep inertia process is maximally influencing neural networks and behavior. See Figure S3 in the Supporting Information for a visualization of all time points under the light condition.

Under the polychromatic short-wavelength-enriched light intervention condition, path length at T1 in the delta band was not significantly different from control (*p* > 0.05; Figure 2A). The clustering coefficient at T1 in the delta band, on the other hand, significantly increased in the light condition compared with control (*t*(10) = −3.04, *p* = 0.01, *g* = 0.54); however, the clustering coefficient was still significantly lower than baseline at T1 in the light condition (*t*(10) = 2.56, *p* = 0.03, *g* = 0.8; Figure 2B). Individual lines show a similar pattern for most participants. There were no significant changes in power between the control and the polychromatic short-wavelength-enriched light conditions for any frequency band, suggesting that polychromatic short-wavelength-enriched light has little impact on power immediately after awakening from SWS (Figure 2C; Figures S2B and S4 in the Supporting Information). Further, Figure S4 shows that power at T1_L_ remains significantly lower than baseline in the alpha and beta bands. Figure S5 in the Supporting Information shows that the clustering coefficient recovered to baseline levels at T2 in both conditions. Just as delta was the only frequency band reflecting sleep inertia under control conditions, the changes observed for delta path length and clustering under the light condition were not observed in other frequency bands, indicating a frequency-specific role of delta following awakening from SWS and in response to polychromatic short-wavelength-enriched light.

### Sleep Inertia Is Characterized by a Global Reduction in Clustering and Region-Specific Rescue With Light

**Figure 3.**
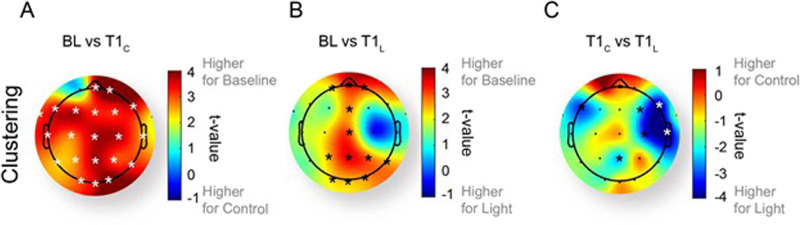
Change in clustering between (A) baseline (BL) and control at T1 (T1_C_), (B) baseline and light at T1 (T1_L_), and (C) control and light at T1 across scalp regions in the delta band. Asterisks represent electrodes with significant difference on a paired *t* test (*p* < 0.05). Electrodes that survived an additional correction for multiple comparisons are highlighted in white (*q* < 0.05). (See Figures S6–S8 in the Supporting Information for an additional analysis across the metrics and frequency bands shown within the main text.)

To understand regional contributions to the changes in the clustering coefficient, we next compared each electrode’s clustering coefficient between the baseline, control, and light conditions in the delta band. Compared with the pre-sleep baseline, in the control condition all but one electrode showed a significant reduction in the clustering coefficient immediately after awakening (*q* < 0.05 after correction for multiple comparisons; Figure 3A). When exposed to light immediately after awakening, there was a trend for reduced clustering across the midline regions of the scalp (Figure 3B), but this did not survive correction for multiple comparisons (*q* > 0.05). When comparing the control and light conditions immediately after awakening (at T1), significant differences were observed in the right hemisphere, with higher clustering in the light intervention condition (Electrode F8: *t*(10) = −3.91; Electrode T8: *t*(10) = −3.95). We also inspected the regional differences in power and degree at each electrode (see Figures S6–S8 in the Supporting Information). Interestingly, in inspecting all of the different effects that we observe at the global average level, we similarly see a variety of electrodes (scattered across the scalp) that may be contributing to this effect.

### Subjective Sleepiness and Behavioral Performance Are Associated With Changes in Small-Worldness

To further attempt to understand these results, we performed correlational analysis at the critical time period for the sleep inertia effects following awakening (T1) and some behavioral and subjective measures collected within the experiment. We concentrate on the control period (T1_C_) and specifically compare global power, average clustering, average path length with the average PVT speed and number of lapses and one subjective measurement of sleepiness (Karolinska Sleepiness Scale; KSS). Interestingly, for this T1_C_ period, global power had no significant associations with either behavioral or subjective data estimated or collected from participants (*p* > 0.05); however, clustering and path length showed several significant effects for both behavioral and subjective ratings. For the subjective data (KSS rating), during the T1_C_ period, we observed a significant positive relationship with path length (*R* = 0.67, *p* = 0.034) within the alpha frequency range, suggesting that higher path length is associated with perceived sleepiness. Also, within the alpha range, clustering coefficient was associated with the KSS rating as well (*R* = −0.64, *p* = 0.048), suggesting that subjects perceived more sleepiness when their brains were displaying lower average clustering coefficient behavior. These results suggest that a link between small-worldness in the brain within the alpha range may be linked to perceived sleepiness.

Behavioral responses, such as PVT speed and lapses, also displayed interesting associations, but within the theta range for clustering coefficient and path length, where higher clustering and lower path length are associated with a faster speed (*R* = 0.7, *p* = 0.024; *R* = −0.7, *p* = 0.022). Lapses show a similar trend where lower clustering and higher path length are associated with more lapses (*R* = −0.67, *p* = 0.034; *R* = 0.67, *p* = 0.032). These results again suggest that metrics associated with small-worldness not only are playing a role in perceived sleepiness, but also are associated with performance fluctuations on a PVT within the theta range.

## DISCUSSION

This is the first study to describe the changes to small-world network dynamics in the waking brain and the impact of polychromatic short-wavelength-enriched light on this profile. Our analyses revealed significant reductions in clustering and increases in path length in the delta band immediately after awakening from SWS at night. Exposure to polychromatic short-wavelength-enriched light attenuated the changes observed in clustering, suggesting a specific regime of segregation and integration driving the waking process. Network analyses, together with our investigation of an intervention and associations with both subjective sleepiness and objective behavioral performance, provide unique insight into the neurophysiological profile of sleep inertia beyond previous approaches.

### Small-Worldness Is Altered During Sleep Inertia

Studies of characteristically small-world activity have shown that small-world properties are higher during sleep, especially in the slower oscillatory schemes, compared with wakefulness (Ferri et al., 2007, 2008). Given this finding, we might expect higher small-worldness features immediately after waking from sleep if there is a slow transition from sleep to wakefulness. We observed, however, a significant reduction in clustering coefficient and increased path length immediately following sleep compared with pre-sleep levels. This change suggests a distinctive scheme towards a more random network with both an observed lower clustering of channels and simultaneously more segregation across them, indicating a relative decrease in small-worldness. Moreover, with fMRI-estimated connectivity, Liu et al. (2014) observed that under conditions of sleep deprivation, the estimated small-worldness increased relative to rested wakefulness, suggesting that the brain may compensate for sleep deprivation via this mechanism. These findings may appear contradictory to what we observed in this study, but important experimental manipulations may provide a key understanding to how small-worldness shapes neural behavior during the critically important state of sleep, including how engaged participants are during a task, and the targeted network, as defined by nuances of the methodology or analysis (e.g., fMRI BOLD vs. EEG power or EEG oscillatory scheme).

Interestingly, with EEG, Koenis et al. (2013) observed a decrease in small-worldness within the alpha band while awake but sleep-deprived, but reported no changes in the delta band. Importantly, these findings were reported while participants were engaged in a reaction time task. Given the similarities with our study design, the differences in our outcomes may reflect a unique profile of the awakening brain that is distinct from the state of sleep deprivation itself. It is notable that immediately after awakening, the primary features of small-worldness describe a unique aspect of brain segregation and integration, independent of both sleep-like features and the influence of sleep deprivation. This finding may indicate that, while participants are engaged in a task, sleep inertia is characterized by neural network reconfigurations that arise due to the disruption of SWS into an awake state. These reconfigurations place the brain network into a state of high segregation.

Inspecting the relationship of behavioral measurements to the graph metrics, PVT performance, as estimated with clustering coefficient and path length, did not show a relationship with global power; however, these metrics showed a relationship with clustering coefficient and path length within the theta band, suggesting that changes in small-worldness are related to behavioral performance fluctuations across subjects. This adds to previous research that has found a significant relationship between performance measurements of this task with fluctuations in power within the theta band within subjects (Gorgoni et al., 2014; Jung et al., 1997) and expands our understanding of network changes associated with this task (Hoedlmoser et al., 2011).

Complementary to the aforementioned study (Koenis et al., 2013), our subjective measurement of sleepiness (KSS) was associated with our metrics of small-worldness within the alpha band, but the alpha band, taking all observed effects into account, did not display a sleep inertia effect. There is a unique relationship between the alpha oscillation and awareness, generally, as it has been shown, in certain cases, to halt the processing of visual information (Mathewson et al., 2009) acting as a gatekeeper of information and even plays a critical role in temporal expectation (Rohenkohl & Nobre, 2011). While these effects represent rapid informational processing, these findings have been generalized to suggest that the alpha band may be required to access knowledge of previous events and grasp contextual (“semantic”) information. Our results seem to provide some general support for this theory, but future research is needed to explore these findings, disentangling these effects on perceived sleepiness, sleep deprivation, and fluctuations within the alpha band.

### Global Power Suggests a Prioritization Scheme May Underlie Sleep Inertia

Previous research seeking to describe the waking brain has focused on EEG power. Oscillations within the brain characterize rhythmic activity of subpopulations of neurons. It has been observed that specific cognitive functions are associated with different oscillatory schemes (Buzsáki, 2006; Neuper & Klimesch, 2006; Nunez & Srinivasan, 2006), where specific frequencies have been associated with top-down processes like executive actions and attention (Engel et al., 2001; Varela et al., 2001), others with motor control or maintenance of sensorimotor behaviors (Engel & Fries, 2010), and others with localized and rapid computations (Fries et al., 2007). Studies of sleep inertia have observed the waking brain exhibiting “sleep-like” attributes like high delta power and low beta power, compared with rested wakefulness (Ferrara et al., 2006; Gorgoni et al., 2015; Marzano et al., 2011). Notably, our baseline testing was conducted following mild sleep restriction, which may have elevated delta power in our baseline measures relative to rested wakefulness (Åkerstedt & Gillberg, 1986), and thus dampened our ability to detect a difference between pre-sleep and post-sleep delta power. This suggests, however, that delta power itself is not a unique signature of the awakening brain. Further, we extended observations of EEG power to show the relative rate of recovery of these frequencies beyond time points previously tested (i.e., beyond 10–25 min post-sleep) and with greater temporal resolution (i.e., more frequent testing points). We observed a marginally significant difference in the global power between baseline and the control condition in the theta band approximately 2 min after awakening and significant differences in global power between baseline and the control condition up to approximately 17 min in the alpha band, and approximately 32 min in the beta band after awakening. While none of these observations were different from each other across post-awakening test bouts, this finding suggests that there is a measurable change in global power in these bands that is sustained for different lengths of time. For example, the recovery to baseline was faster for theta frequencies and longest for beta power, taking at least 30 min to return to pre-sleep levels. These observations support and extend findings from others who also reported reductions in alpha and beta activity immediately following awakening (Ferrara et al., 2006; Gorgoni et al., 2015; Marzano et al., 2011), but did not report the subsequent time course of recovery.

Our observations, overall, suggest that the broader organization of the brain may underlie the slower dissipation rate of impairment typically observed after approximately 15 min that continues across the next hour (Jewett et al., 1999; Wertz et al., 2006). These findings suggest that cognitive functions associated with slower oscillations (i.e., theta band, 3–7 Hz) have a more rapid recovery than those associated with faster oscillations (i.e., beta band 13–25 Hz). In addition to these findings, correlational analyses with PVT behavioral performance suggest a critical role of the theta band small-worldness and its associations with behavioral performance.

Taken together with the findings that specific cognitive functions are associated with different oscillatory schemes (Buzsáki, 2006; Neuper & Klimesch, 2006; Nunez & Srinivasan, 2006)—slower frequencies generally with top-down processes like executive actions and attention (Engel et al., 2001; Varela et al., 2001); higher frequencies with motor control or maintenance of sensorimotor behaviors (Engel & Fries, 2010); and even higher frequencies with localized, specific, and rapid computations (Fries et al., 2007)—it follows that sleep inertia is also characterized by different time courses in cognitive subsystem recovery (e.g., working memory task vs. simple math task; Achermann et al., 1995; Jewett et al., 1999). It may also signal a prioritization scheme of the waking brain, from high-level executive functions to motor coordination; however, further research is necessary to understand the potential differences in cognitive system recovery.

### Long-Range Connections Orchestrating Local-Global Operations Are Uniquely Disrupted Within the Brain Shortly After Awakening

The suggested prioritization scheme in power, in addition to the delta band specificity in network changes, could suggest how this prioritization scheme is implemented in the brain. Oscillations emanating from the brain, as measured via EEG, are a consequence of short- and long-range connections within the brain that coalesce to support cognition (Buzsáki et al., 2013). Slower oscillations often represent the coordination of distal regions of the cortex that modulate higher frequency oscillations within the brain (Arnulfo et al., 2015; Bragin et al., 1995; Buzsáki & Schomburg, 2015; Buzsáki & Wang, 2012; Canolty et al., 2006; Chrobak & Buzsáki, 1998). In other words, oscillatory activity and the associated cognitive functions may be understood as a consequence of the ever-present need and importance of global coordination of local processes (Bressler & Kelso, 2001). This local-global coordination of neural activity is critical to a variety of cognitive processes (Sauseng & Klimesch, 2008), is the hallmark of several diseases (Schnitzler & Gross, 2005), and is an organizing principle of brain activity that has been suggested to be foundational even across multiple species (Buzsáki et al., 2013). It has even been suggested that the oscillatory synchronization across brain sites is related to brain size (i.e., distance between coordinated regions; Nunez et al., 1978; Valdés-Hernández et al., 2010), suggesting that this coordination across distal and proximal brain regions may not only underlie cognitive processing but also may be associated with short- and long-range connections within the brain. Within this framework, our global power results might suggest that long-range connections, as indicated by slower oscillations (delta and theta) and large-scale cortical integration (Bruns & Eckhorn, 2004), recover more rapidly than the higher frequency bands (e.g., more localized computation; Buzsáki & Wang, 2012). While global power in lower frequency bands is influenced by ensemble synaptic action across long ranges and averaged across all channels, our graph theoretic analysis is derived from the statistical dependencies between nodes, specifically estimating phase-based relationships between different channels. In other words, while global power in slower oscillations is sensitive to both amplitude and phase-based relationships aggregated across the long-range connections within the brain, our graph theoretic results are narrowly sensitive to phase-based coordination within longer connections, or the *communication structure* rather than fluctuations in synchronous neural activity. Taken together, our results display a prioritization of longer range—perhaps higher cognitive—coordinated activity after awakening, while simultaneously increasing communication efficiency across them. Future research may investigate the role of higher segregation and long-range integration of networks during this process.

### Polychromatic Short-Wavelength-Enriched Light Serves as an Intervention to Mitigate Neural Effects of Sleep Inertia

We also studied an intervention condition in which participants were exposed to a polychromatic short-wavelength-enriched light immediately after being awakened from SWS and throughout the 1-hr testing period. Light, particularly bright, short-wavelength-enriched light, is known to have acute alerting properties when administered under conditions of sleep deprivation and is particularly effective when used during the biological night (Lok et al., 2018; Souman et al., 2018). EEG studies have shown that acute exposure to short-wavelength-enriched light at night during continuous wakefulness reduces delta-theta power (0.5–5.5 Hz, a biomarker of sleepiness; Lockley et al., 2006; Phipps-Nelson et al., 2009; Rahman et al., 2014) and increases alpha and high-alpha power (9.5–10.5 Hz, a biomarker of alertness; Cajochen et al., 1998; Lavoie et al., 2003; Lockley et al., 2006; Rahman et al., 2014).

Recently, we reported the effects of light during the sleep inertia period following nocturnal awakenings. Our study showed that light modestly improved performance on a psychomotor vigilance task as well as subjective outcomes such as alertness and mood (Hilditch et al., 2022). In the current paper, we report the potential mechanisms underlying these neurobehavioral effects of light during the sleep inertia period. In contrast to previous findings during continuous wakefulness at night, we observed only modest, regional changes in power with light exposure during the sleep inertia period (see the Supporting Information). This contradiction in findings may suggest that light acts through a different mechanism during the sleep inertia period.

We observed that the significant decreases in clustering in the delta band following awakening in the control condition were attenuated with exposure to polychromatic short-wavelength-enriched light. Thus, our observed effects of light on the awakening brain appear to counteract the unique hallmark of the sleep inertia period illustrated by our novel network analysis. Taken together with our lack of changes in global power with polychromatic short-wavelength-enriched light exposure, this finding strengthens the specificity of the long-range communication aspect of our results. During the sleep inertia period, polychromatic short-wavelength-enriched light may help to restore or protect against the dis-coordination of long-range communication within the brain. This manipulation of delta networks may represent a mechanism through which polychromatic short-wavelength-enriched light exposure influences neurophysiological properties to improve alertness, mood, and performance immediately after awakening (Hilditch et al., 2022). To our knowledge, no previous studies have evaluated the effect of a light stimulus on these network dynamics in different brain states; our study is the first to describe and tentatively interpret these effects.

### Methodological Considerations and Limitations

Although our study involved a randomized, within-subject, crossover design with frequent testing points and 32-channel EEG, it is not without limitations. First, our study did not include a measure of melatonin to explore whether manipulation of its secretion was a potential contributing factor to the differences observed in the polychromatic short-wavelength-enriched light condition. We assume based on prior literature, however, that melatonin was suppressed for the duration of the light exposure (Lewy et al., 1980; Lockley et al., 2006; Zeitzer et al., 2000) and, therefore, may act as a mechanism for the acute effects of light. Second, while a strength of our study is that we controlled the sleep stage at awakening, this was traded for potential changes in circadian sleep pressure between the two awakenings. The order of condition was randomized to limit any differences, and the mean time between awakenings was 90 min (Hilditch et al., 2022), so we do not expect large differences from this design. Further, the likelihood of a circadian effect in the current study is low as we only observed the reduction in clustering and path length immediately after awakening, with a rapid return to baseline levels, which is not characteristic of a circadian effect. We acknowledge that we are unable to directly disentangle the relative contributions from, or effect of light on, the three sleep processes (homeostatic, circadian, inertia) in the current study. However, our first testing point (T1 at 2 min post-awakening) is a robust proxy for sleep inertia. Importantly, we have, for the first time, demonstrated the neurophysiological profile of the awakening brain and the effect of polychromatic short-wavelength-enriched light exposure following awakening from SWS during a nocturnal sleep episode, which is a common scenario for on-call and emergency service workers.

### Conclusions

The current study extends prior research investigating the waking brain by building a more comprehensive description of the neurophysiological profile of the brain following awakening from SWS at night. Our results suggest that long-range network communication within the brain is crucial to the waking process and, further, that the brain may prioritize these long-range connections, adding to the evolutionary importance of the coordination of local and global activity within the brain. Moreover, the addition of a within-subject assessment of the effects of polychromatic short-wavelength-enriched light exposure provides more insight into our understanding of sleep inertia, adds a causal aspect to our findings, and suggests how we might mitigate its effects to improve alertness and performance in safety-critical scenarios.

## MATERIALS AND METHODS

### Participants

Twelve healthy young adults participated in the study, having met the following inclusion criteria: healthy (General Health Screening Questionnaire, personal physician’s permission to participate, approval from onsite physician upon review of urinalysis and blood work screening); normal sleepers (Pittsburgh Sleep Quality Index ≤ 5; no self-reported sleep problems; habitual sleep of 7–9 hours); no shiftwork or travel > 3 time zones in the past 3 months (self-report); free of illicit substances and nicotine (urine toxicology screen); and free of alcohol during the study period (breathalyzer). All participants provided written informed consent. The protocol was approved by the NASA Ames Research Center Institutional Review Board (HRII-371 and HRII-20-04). One participant’s dataset was incomplete; therefore, results presented here reflect a sample population of *n* = 11.

A priori power calculations were based on anticipated changes in our primary outcome measure, PVT performance, the results of which are presented elsewhere (Hilditch et al., 2022). The PVT used in this study is a 5-min reaction time task used to measure vigilant attention (Loh et al., 2004; Roach et al., 2006). Participants are required to monitor a screen and respond to stimuli presented at random intervals as soon as possible by pressing a button with their dominant thumb. Based on previous studies in similar populations, we expected that the effect size for the change in PVT during sleep inertia would be approximately 0.75. Using these assumptions, we estimated that we would need 10 participants to detect a change in performance with 80% power at an alpha level of 0.05. Prior studies investigating sleep interventions including similar sample sizes have observed significant and equivalent changes in both reaction time test and EEG connectivity outcome measures (Koenis et al., 2013).

### Procedure

The results presented here come from a 2-week study. Here we report a 1-week within-subject, crossover intervention study with the presentation order of intervention randomized by sex. A summary of relevant results from the alternate week are presented in the Supporting Information.

**Figure 4.**
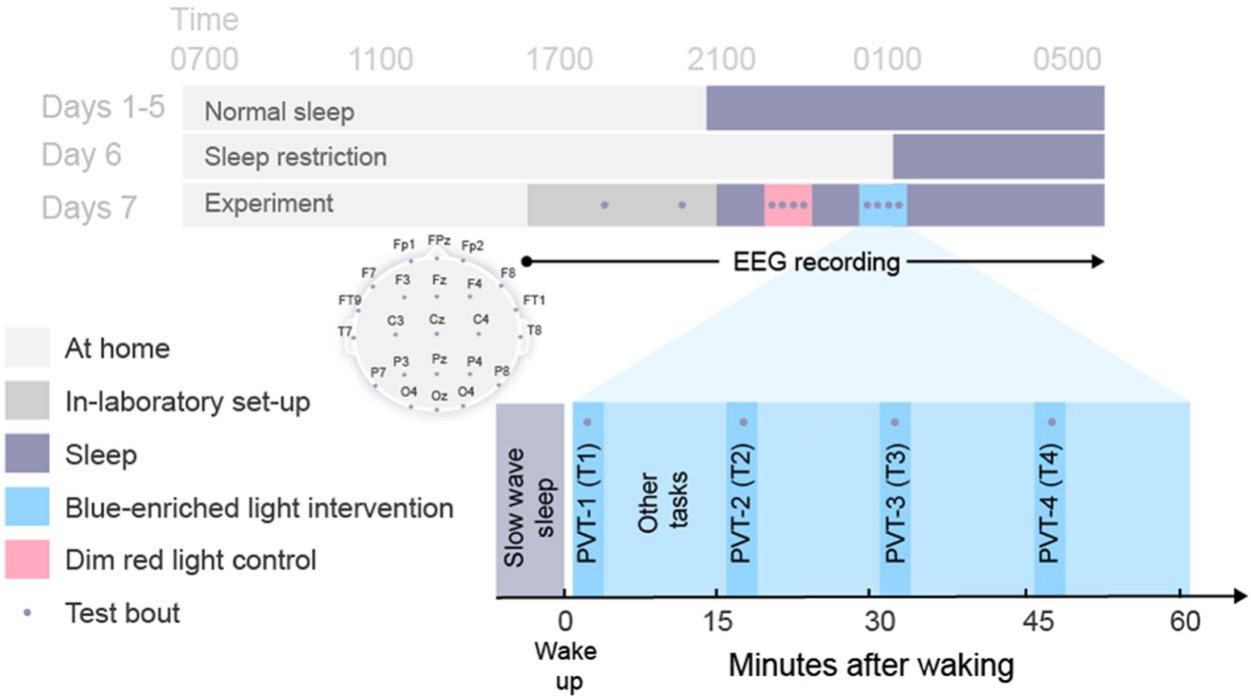
Protocol schematic. Light gray shading indicates wakefulness during the at-home portion of study. Dark gray shading indicates in-laboratory pre-sleep activities including baseline testing (•). Black shading indicates sleep opportunities (<0.3 lux). Blue and red shading indicate intervention and control sleep inertia testing periods, respectively. Inset shows electrode montage and post-awakening test bouts. Clock times shown are approximate and varied depending on habitual sleep-wake times and appearance of slow wave sleep periods.

Participants were required to follow a fixed sleep-wake schedule based on habitual sleep timing for the 6 nights leading up to the in-laboratory visit (see Figure 4). Following a night of at-home sleep restriction (5 hr), participants were brought into the sound attenuated, light- and temperature-controlled laboratory for pre-sleep procedures that included task familiarization, electrode setup, and baseline tests prior to overnight observation and testing. Baseline EEG measures were taken during a PVT performed 2.5 hr before habitual bedtime.

At the participant’s habitual bedtime, all lights were turned off (<0.3 lux) and the participant was instructed to sleep. EEG was monitored during the sleep period to identify slow wave sleep (SWS) stages (Stage 3 and 4; Rechtschaffen & Kales, 1968). Participants were awakened after a minimum of 5 consecutive min of SWS. Immediately upon awakening, a dim red ambient light was illuminated in the room. In the intervention condition only, at 1 minute post-awakening, a polychromatic short-wavelength-enriched light was illuminated and remained on for the hour of testing. The dim red ambient light remained on during the testing period in both conditions. A 5-min PVT was performed four times (T1, T2, T3, and T4 at +2, +17, +32, and +47 min after the awakening, respectively), during which EEG was recorded. At the end of the testing period, all lights were turned off and the participant was instructed to return to sleep. EEG was monitored again to identify the next period of 5 consecutive min of SWS, at which time the participant was awoken again and exposed to the opposite condition (polychromatic short-wavelength-enriched light or control). Following the second testing period, all lights were turned off and the participant was instructed to sleep until they were awakened at their habitual wake time.

### Intervention

A 12″ × 24″ canvas of light-emitting diodes (Circadian Positioning Systems, Inc., Newport, RI) was positioned at 15 degrees to the horizontal angle of gaze and approximately 56 cm away from the participant. Light levels during the intervention and control conditions were confirmed via Spectroradiometer ILT950 (International Lighting Technologies, Peabody, MA). Illuminance, irradiance, equivalent daylight (D65) illuminance (EDI), and peak spectra during the intervention were 242.77 lux, 0.95 W/m^2^, 338.03 melanopic lux, and 456 nm, respectively, measured at the angle of gaze. An ambient dim red light served as the control (0.26 lux, 0.00 W/m^2^, 0.10 melanopic lux, 714 nm).

### EEG Analysis

#### Preprocessing.

EEG was recorded during the baseline and post-awakening testing periods using BrainVision 32-channel caps with sintered Ag/AgCl electrodes (Brain Products GmbH, Munich, Germany) and BrainVision Recorder software (Brain Products GmbH, Munich, Germany) recording at a sampling rate of 500 Hz. Additional electrodes included bipolar horizontal electrooculogram (EOG: left/right placed on the outer canthus of the eye 1 cm above and below the horizon, respectively), and submental electromyogram (EMG), for standard monitoring with sleep periods. For visualization while the experimenter monitored the EEG, a 70 Hz high-pass filter in conjunction with a notch filter at 60 Hz was used online so that the experimenter could easily determine whether the participant was in SWS and primed for an awakening. After the recording sessions, the raw, unfiltered EEG recordings were then subjected to a thorough artifact editing scheme offline. After a preliminary filtering of the raw EEG data, using a third-order zero-phase bandpass Butterworth filter (0.5–50 Hz) in EEGLAB (Delorme & Makeig, 2004), the EEG data were subjected to artifact subspace reconstruction (ASR; Kothe & Makeig, 2013; Kothe & Jung, 2016; Mullen et al., 2015). This method removes extremes in data using a time-evolving blind source separation method; importantly, this method has been shown to be particularly resilient to artifact encountered in real-world scenarios (Mullen et al., 2013). To deploy ASR on the dataset, we first created a “clean” reference signal from each participant’s EEG data by concatenating EEG segments that were at least 1,000 ms long with amplitude below 100 μV, most likely not contaminated by artifacts due to muscle activity following the awakening. Following the creation of the reference signal, ASR was then used to clean the EEG that contained large fluctuations greater than 5 standard deviations beyond the reference signal (in 500-ms chunks). Finally, EEG data from the beginning to the end of each PVT (approximately 5 min each) were identified and then filtered via a third-order zero-phase bandpass Butterworth filter within the frequency bands of interest (delta: 1–3 Hz, theta: 4–7 Hz, alpha: 8–12 Hz, beta: 15–25 Hz). This produced four sets of continuous time courses for each of the time segments following the awakening (T1, T2, T3, T4) in addition to the baseline pre-sleep time period (BL) for each participant.

#### Global power spectral density.

Power spectral density (PSD) was estimated using a standard approach of Welch’s average modified periodogram method of spectral estimation (Welch, 1967) in MATLAB (MathWorks, Inc.). The log-transformed PSD values were then standardized for each electrode before analysis by mean-centering each channel and dividing by standard deviation across the entire frequency range (0 to 250 Hz). To represent a given frequency band of interest, standardized PSD values were averaged over the frequency range of that band. For the sake of simplicity, we use the term *power* to represent the standardized PSD values and the term *global power* to represent the average standardized PSD across the scalp for a given frequency band of interest.

#### Network connectivity.

To estimate the functional network connectivity between EEG sensors, we computed the pairwise weighted phase lag index (wPLI), which is known to be highly sensitive to linear and nonlinear interactions (Imperatori et al., 2019); it is well established that phase-based measurements of connectivity are less susceptible to nuisance artifacts (Lau et al., 2012). wPLI belongs to a suite of phase-based measurements often deployed with EEG signals to mitigate the effects of volume conduction (Vinck et al., 2011); however, amplitude-based connectivity measurements have been shown to display robust effects in particular systems (e.g., motor; Wei et al., 2021). Importantly, wPLI, when used as the backbone for graphical analyses, has been shown to produce highly reproducible graph metrics within subjects while simultaneously capturing individual differences between subjects (Hardmeier et al., 2014). Thus, within a participant, we calculated the wPLI matrix for all the time points and frequency bands by using the band-wise filtered EEG activity for the entire 5-min trial epoch. This produced one weighted and undirected connectivity matrix for T1, T2, T3, T4, and the baseline pre-sleep time period.

#### Network analysis.

Three network measurements were estimated on the wPLI matrices, including clustering coefficient, path length, and (in the Supporting Information) degree. These common metrics have been used to describe properties of many complex systems including a variety of biological, social, and other phenomena (Bassett & Sporns, 2017; Collins & Chow, 1998; Seaton & Hackett, 2004) and are often evoked when describing small-world phenomena (Collins & Chow, 1998). Here, we use clustering coefficient and path length to describe network changes related to small-worldness/randomness of the network and degree as a visualization of changes in network connectivity.

Specifically, clustering coefficient (*C*) estimates the tendency of a node’s neighbors within a network to also be linked, and may be mathematically described from a connectivity matrix, *W* (here estimated via wPLI; Onnela et al., 2005; Rubinov & Sporns, 2010).

$$C_{i}=\frac{2}{K_{i}\left(K_{i}-1\right)}\sum_{j,k}{\left(w_{i,j}w_{j,k}w_{k,i}\right)}^{1/3},$$ (1)

where *w*_*i*,*j*_ represents an element of the connectivity matrix *W* implying the strength of connectivity between nodes *i* and *j*. *C*_*i*_ and *K*_*i*_ represent the clustering coefficient and degree for node *i*. Degree of a node is defined as the sum of all the edge weights connected to it and is a general representation of a node’s connectivity across the network:

$$K_{i}=\sum_{j}w_{i,j}.$$ (2)

Path length, on the other hand, estimates the number of edges, on average, that must be traversed to connect any two nodes within a network. If *d*(*i*, *j*) represents the shortest path through edges between nodes *i* and *j*, path length *λ* is given by (Rubinov & Sporns, 2010)

$$\lambda =\frac{1}{n\left(n-1\right)}\sum_{i\neq j}d\left(i,j\right),$$ (3)

where *n* is the total number of nodes. In general, the distance between two nodes with strong connectivity is lower than the distance between nodes with relatively weaker connectivity. Therefore, to estimate *d*(*i*, *j*), we used (1 − *w*_*i*,*j*_), and then the path length was calculated using Brain Connectivity Toolbox function *charpath.m*.

#### Neurobehavioral analysis.

As a final analysis, we explored the associations between three behavioral metrics and the global neural metrics found to be susceptible to the state of sleep inertia. The three behavioral metrics were subjective sleepiness (KSS), and objective cognitive performance as assessed by mean PVT speed (1/reaction time, RT) and number of PVT lapses (RT > 500 ms). Pearson’s correlation coefficient was obtained in MATLAB (MathWorks, Inc.) via the *corr.m* function (see the Results section and Supporting Information Figure S9).

#### Analysis and statistics.

Our primary analysis explored the network configuration of the awakening brain under control conditions as compared with the pre-sleep baseline period across four frequency bands (described above). Our secondary analysis explored the impact of polychromatic short-wavelength-enriched light on this network profile by comparing conditions (control and light). Specifically, to assess any changes in the metrics of power and network connectivity we used paired *t* tests (in MATLAB, MathWorks, Inc.) with a significance criterion of *p* < 0.05. Therefore, we compared pre-sleep baseline period and each of the post-sleep test bouts for both control and light using paired *t* tests for all the frequencies of interest. Additionally, we compared the respective test bouts between conditions (control and light) to explore the impact of polychromatic short-wavelength-enriched light across frequencies. Significance criterion was applied on uncorrected *p* values. Where appropriate for multiple comparisons, false discovery rate was used with a *q* threshold set to 0.05 within a given frequency band. To estimate the effect size of significant findings, we computed the Hedges’ *g*, defined as the difference between the sample means normalized by the pooled standard deviation. Hedges’ *g* was used to express the effect size here, as it better characterizes the effect size with samples lower than 20 (Lakens, 2013).

#### Visualization.

All figures, boxplots, and topographic plots were created in MATLAB (MathWorks, Inc.) with common core functionality from MATLAB and some additional functions with EEGLAB (Delorme & Makeig, 2004). Then, they were imported into Adobe Illustrator (version 25.3) and combined into panels for visualization.

## ACKNOWLEDGMENTS

This work was supported in part by the Naval Medical Research Center’s Naval Advanced Medical Development Program (MIPR N3239820WXHN007) and the NASA Airspace Operations and Safety Program, System-Wide Safety. This work was also supported in part through mission funding from the US DEVCOM Army Research Laboratory (ARL). K. B. also acknowledges support from ARL through Cooperative Agreement Number W911NF-16-2-0158. The views and conclusions contained in this document are those of the authors and should not be interpreted as representing the official policies, either expressed or implied, of the ARL or the US government. The authors would like to thank Aditi Periyannan for her help with figure design and layout. The authors would also like to thank all the participants who volunteered their time for this study.

## DATA AVAILABILITY

Data are accessible upon reasonable request as far as allowed by the data-sharing policy and guidelines established by NASA Ames Research Center.

## SUPPORTING INFORMATION

Supporting information for this article is available at https://doi.org/10.1162/netn_a_00272.

## AUTHOR CONTRIBUTIONS

Cassie J. Hilditch: Conceptualization; Data curation; Funding acquisition; Investigation; Methodology; Writing – original draft; Writing – review & editing. Kanika Bansal: Data curation; Formal analysis; Methodology; Visualization; Writing – original draft; Writing – review & editing. Ravi G. Chachad: Data curation; Investigation. Lily R. Wong: Investigation; Writing – review & editing. Nicholas G. Bathurst: Project administration; Writing – review & editing. Nathan H. Feick: Investigation; Methodology. Amanda Santamaria: Methodology; Writing – review & editing. Nita L. Shattuck: Funding acquisition; Writing – review & editing. Javier O. Garcia: Data curation; Formal analysis; Methodology; Writing – original draft; Writing – review & editing. Erin E. Flynn-Evans: Conceptualization; Funding acquisition; Investigation; Methodology; Supervision; Writing – review & editing.

## FUNDING INFORMATION

Cassie Hilditch and Erin Flynn-Evans, NASA Airspace Operations and Safety Program, System-Wide Safety. Kanika Bansal and Javier Garcia, US DEVCOM Army Research Laboratory. Kanika Bansal, US DEVCOM Army Research Laboratory, Award ID (Cooperative Agreement Number): W911NF-16-2-0158. Nita Shattuck, Naval Medical Research Center’s Naval Advanced Medical Development Program, Award ID: MIPR N3239820WXHN007.

## TECHNICAL TERMS

Sleep inertia: The brief period of impaired alertness and cognitive performance experience immediately after waking.
Homeostatic sleep pressure: The increase in sleep propensity with increasing sleep loss or extended wakefulness.
Circadian rhythm: The change in sleep propensity across a near-24-hr cycle as determined by a circadian pacemaker (suprachiasmatic nucleus).
Graph theoretical framework: A mathematical framework used to describe the relationships between connected objects.
Clustering coefficient: Estimates the tendency of a node’s neighbors within a network to also be linked.
Path length: Estimates the number of edges, on average, that must be traversed to connect any two nodes within a network.
Small-world network: A network lying in between a random network and a regular lattice, reflecting intermediate values of both path length and clustering.
