# Erratum: Reconfigurations in brain networks upon awakening from slow wave sleep: Interventions and implications in neural communication

**DOI:** 10.1162/netn_x_00359

**Published:** 2024-04-01

**Authors:** Cassie J. Hilditch, Kanika Bansal, Ravi Chachad, Lily R. Wong, Nicholas G. Bathurst, Nathan H. Feick, Amanda Santamaria, Nita L. Shattuck, Javier O. Garcia, Erin E. Flynn-Evans

**Affiliations:** Fatigue Countermeasures Laboratory, Department of Psychology, San José State University, San José, CA, USA; Department of Biomedical Engineering, Columbia University, New York, NY, USA; US DEVCOM Army Research Laboratory, Humans in Complex Systems Division, Aberdeen Proving Ground, MD, USA; Fatigue Countermeasures Laboratory, Human Systems Integration Division, NASA Ames Research Center, Moffett Field, CA, USA; Cognitive and Systems Neuroscience Research Hub, University of South Australia, Adelaide, SA, Australia; Operations Research Department, Naval Postgraduate School, Monterey, CA, USA

After publication of our paper *Reconfigurations in brain networks upon awakening from slow wave sleep: Interventions and implications in neural communication*, we noticed an error in our condition labels that required some revision of figures and text and some new interpretations throughout the manuscript. The error was a consequence of the double-blind nature of the original experimentation which contained two interventions: (1) a blue-enriched light intervention, as originally mentioned; and (2) an olfactory odorant condition. In review of previous analyses and presentations of the data, there was a time when non-descriptive labels (e.g., “A”, “B”) were accidentally swapped, indicating the wrong intervention label. This label was then subsequently propagated throughout the analysis and into the manuscript. This error was discovered in a re-analysis of the data and is corrected in the revised manuscript.

While this error does not change the overall novel understanding of the ‘sleep inertia’ state as well as ‘interventions’ generally, there are several points in the manuscript where some interpretation of the light and odorant interventions required some additional text or some minor changes. We have edited our interpretation of the data relative to the odorant and added the correct light data for completion, finding that the olfactory stimulus (peppermint) appeared to alleviate neural consequences of sleep inertia where blue-enriched light did not, at least within a narrow band of EEG oscillations (i.e., delta). While not the primary finding of the manuscript, we now conclude the manuscript with a short discussion on potential mechanisms to this interesting finding.

The following is a more detailed list of changes made to the main manuscript:

- The intervention initially discussed as polychromatic short-wavelength-enriched light is now correctly identified as peppermint odorant. Throughout the manuscript, this identification has been corrected. A subscript “O” is used for the odorant intervention which is now used instead of “L” in Figures 2 and 3.
- For transparency, the details of both the interventions are now added to the paper in the Materials and Methods section; and the Results section is modified to include that no significant effect of the light intervention was observed on sleep inertia signatures. In this regard, the Results subsection previously titled “Polychromatic short-wavelength-enriched light exposure at awakening attenuates neural network changes associated with sleep inertia in the delta band” is now titled “Exposure to an odorant at awakening attenuates neural network changes associated with sleep inertia in the delta band”. The Results subsection previously titled “Sleep inertia is characterized by a global reduction in clustering and region-specific rescue with light” is now titled “Sleep inertia is characterized by a global reduction in clustering and region-specific rescue with an odorant intervention”.
- We discovered that while the condition labeling of intervention for neural data was swapped, it remained correctly identified for the behavioral data. Therefore, we repeated the analysis comparing our neural metrics and behavioral measures. We did not observe any relationship between PVT outcomes and neural metrics with the correct labeling of conditions. We observed that the global power within the beta band was negatively correlated with the subjective measurement of sleepiness (KSS) (R = −0.7, p = .017). Please see the Supporting Information Figure S9 for these results. To incorporate these new findings, the Results subsection previously titled “Subjective sleepiness and behavioral performance are associated with changes in small-worldness” is now titled “Subjective sleepiness, behavioral performance, and neural metrics”.
- Within this same context as above, the Discussion subsection titled “Small-worldness is altered during sleep inertia” has been modified to remove any discussion relating small-worldness and behavioral features that was included in the previous version.
- The Discussion subsection previously titled “Polychromatic short-wavelength-enriched serves as an intervention to mitigate neural effects of sleep inertia” is now titled “An odorant serves as an intervention to mitigate neural effects of sleep inertia”. To explain the new findings regarding peppermint odorant intervention, new text and citations have been added to this section.
- As we did not observe the effect of the light intervention on sleep inertia, in the Discussion subsection titled “Methodological Considerations and Limitations”, the text discussing the potential limitations of our interpretation of the effect of light intervention is now removed.

The list of changes made to the Supporting Information:

- Figures S2–S8 have been modified to indicate correct intervention, i.e., odorant, by changing the subscript from L to O where applicable.
- Figure S9 has been replaced with new behavioral findings as discussed in the main paper and described above.

