# Supplementary material for: Reconfigurations in brain networks upon awakening from slow wave sleep: Interventions and implications in neural communication
Source: Netw Neurosci. 2023 Jan 1;7(1):102–21. doi: 10.1162/netn_a_00272 (PMC10270716; doi:10.1162/netn_a_00272)
Supplement: Supplementary file 1 [file netn-7-1-102-s001.pdf]

Hilditch, C. J., Bansal, K., Chachad, R., Wong, L. R., Bathurst, N. G., Feick, N. H., Santamaria, A., Shattuck, N. L., Garcia, J. O. & Flynn-Evans, E. E. (2023). Supporting information for "Reconfigurations in brain networks upon awakening from slow wave sleep: Interventions and implications in neural communication." *Network Neuroscience*, *7*(1), 102–121. https://doi.org/10.1162/netn_a_00272

Supplemental Materials

Reconfigurations in brain networks upon awakening from slow wave sleep: Interventions and implications in neural communication

Hilditch CJ*, Bansal K*, Chachad R, Wong LR, Bathurst NG, Feick NH, Santamaria A, Shattuck NL, Garcia JO**, Flynn-Evans EE**

*Co-first authors; **Co-senior authors

Table S1: Participant sleep history (*n* = 11)

	Mean	SD	Range
At-home sleep duration (h)			
Nights 1-5	7.2	0.6	6.4 – 8.2
Night 6	4.4	0.3	3.8 – 5.1
Questionnaires			
PSQI	2.1	1.2	0 – 4
FSS	26.7	7.0	16 – 39
MEQ	55.5	6.9	45 – 64

Note: Sleep variables estimated by actigraphy: sleep duration = sleep period minus wake after sleep onset. Range and SD are based on participant means. SD = standard deviation; h = hour; PSQI = Pittsburgh Sleep Quality Index; FSS = Fatigue Severity Scale; MEQ = Morningness-Eveningness Questionnaire.

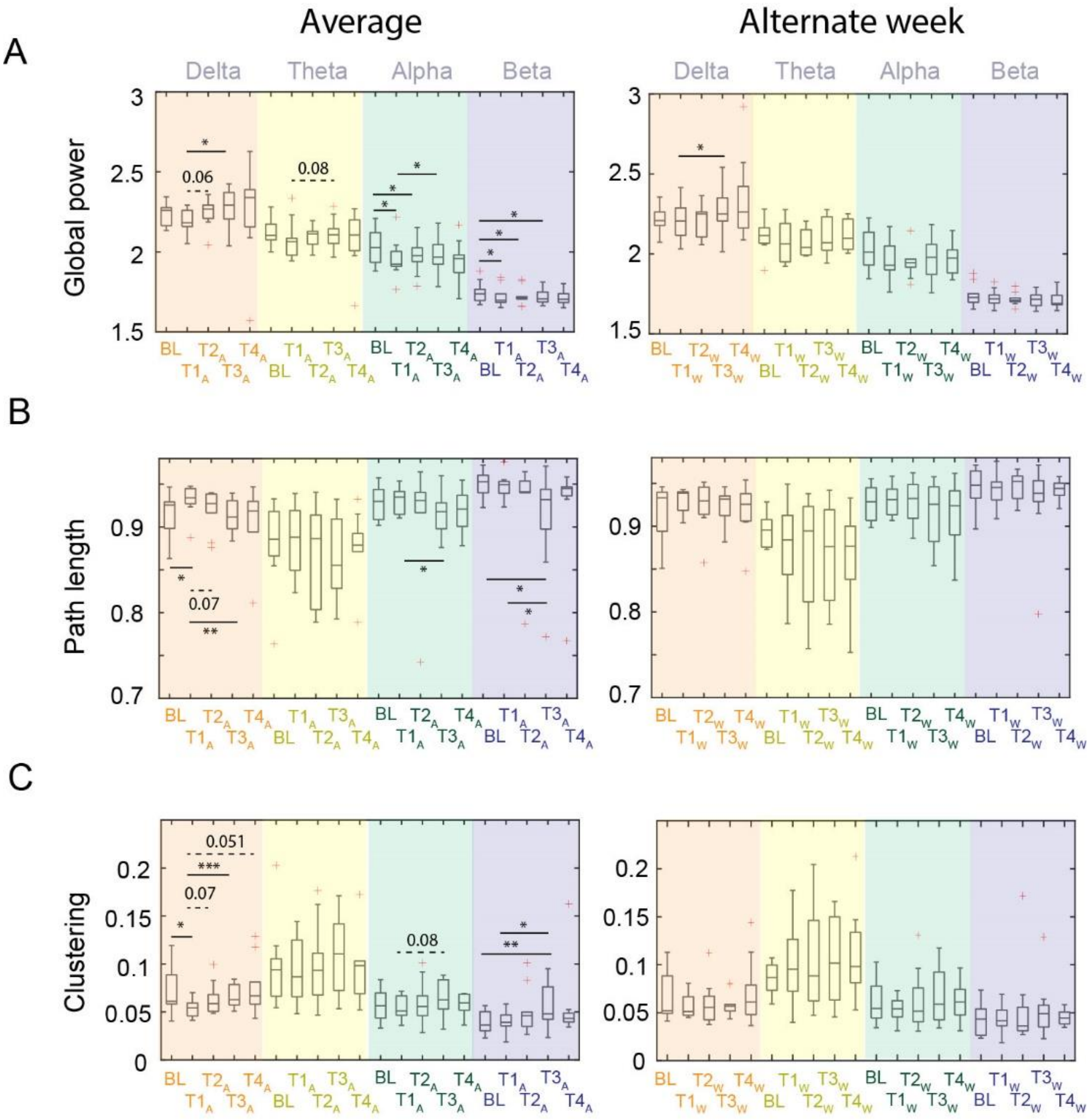

Figure S1: (A) Comparison for power, and (B) - (C) brain network properties across test bouts for each frequency band under dim, red light conditions. Left column displays averaged data from testing sessions in both weeks; right column displays data collected one week apart from the control condition. BL = baseline, $T\#_A$ = Test bout # for data averaged across weeks, $T\#_W$ = Test bout # during the alternate week. Asterisks represent significant difference on a paired *t*-test without any further correction applied such that *$p < .05$; **$p < .01$; ***$p < .001$. Dashed line denotes marginally significant difference (p as indicated).

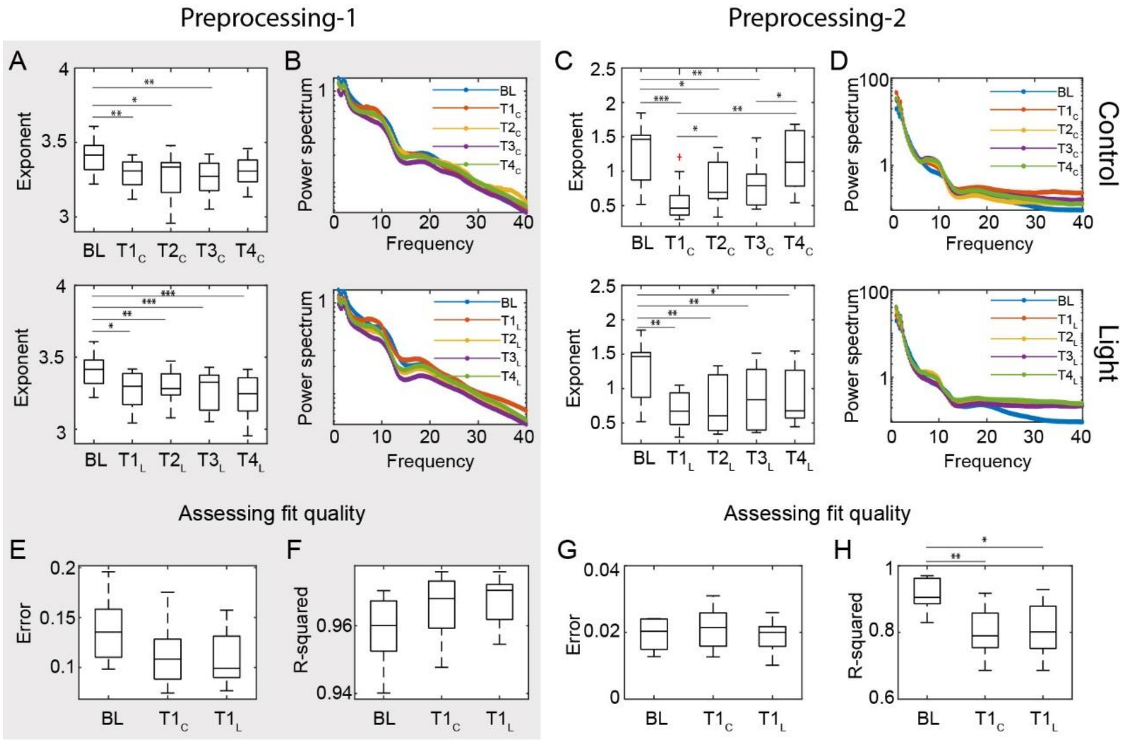

Figure S2: Aperiodicity and Spectral Means. Recent work has attempted to disentangle the aperiodic and periodic signals in human EEG (Donoghue et al., 2020). **Preprocessing-1 (left):** To test whether the aperiodicity in EEG was contributing to the effects in sleep inertia we used the FieldTrip (Oostenveld et al., 2011) and the Fitting Oscillations and One Over f (1/f; Donoghue et al., 2020) set of analytical tools in Matlab (Mathworks, Inc.). Unlike *Preprocessing-2* (right), the analysis here used the same preprocessing steps as in the original analysis that included band-pass filtering and artifact subspace reconstruction (see Methods in the manuscript). With these data, using primarily default or suggested parameters for the FOOOF pipeline, the processed data were chunked into 2-second windows (50% overlap) and submitted to the aperiodic analysis with a multitaper frequency transformation (‘mtmfft’). (A) Average exponent from the FOOOF analysis that parameterizes the spectral fit and is a proxy of the aperiodic component in the spectra. Significant changes are highlighted. Asterisks represent significant difference on a paired *t*-test without any further correction applied such that *$p < .05$; **$p < .01$; ***$p < .001$. (B) To add to this analysis, we also plot the mean power spectra across the different temporal periods including the pre-sleep baseline period (BL) with and without blue-enriched light intervention, without error bars to clearly see the important regions of the spectra that may show differences. Importantly, unlike the data within the main paper, we do not normalize the spectral power here

but do plot on a logarithmic scale. To assess the fit quality of the FOOOF pipeline, error (E) and variance explained (R-squared, F) are also plotted. Student's *t*-test (paired) were used to explore any differences between the fits for each condition including the pre-sleep baseline (BL) and first test bout for control ($T1_C$) and the blue-enriched light condition ($T1_L$). Quality of fit tests were non-significant ($p > .05$). Interestingly, this analysis showed uncorrected differences between the BL period and each test bout except for $T4_C$, suggesting a fundamental difference between aperiodic components pre-sleep to after awakening; however, the analysis did not reveal the sleep inertia effects observed in the primary analysis of graph metrics. **Preprocessing-2 (right):** The analysis here was implemented identically to *Preprocessing-1* (left), except with one key difference: the FOOOF pipeline detailed above was completed on a slightly different preprocessed dataset. Due to the susceptibility of this analysis to have skewed results after broad band-pass filtering, only one preprocessing step was completed on the raw EEG data such that line noise was removed via spectral interpolation in FieldTrip (*ft_preproc_dftfilter.m)* with the algorithm described by Leske & Dalal (2019). Interestingly, different from *Preprocessing-1*, we observed what appears to be an effect of sleep inertia where $T1_C$ is different from the pre-sleep baseline (BL) and $T2_C$ ($p < .05$) and then this effect is attenuated by blue-enriched light. Assessing the fit of this aperiodic analysis, though, we also observed a significant difference between BL and $T1_C$ and between BL and $T2_C$ ($p < .05$). Thus, the observed sleep inertia effect may not solely reflect the sleep inertia state and instead may be susceptible to nuisance variables. Overall, the sleep inertia effects within the primary analysis do not appear to be driven by the aperiodic components; however, the subsequent analysis shows promise that aperiodicity could play a role in sleep inertia.. A higher-powered dataset and an exploration of the noise associated with sleep inertia will be needed to fully explore the aperiodicity components underlying the awakening process.

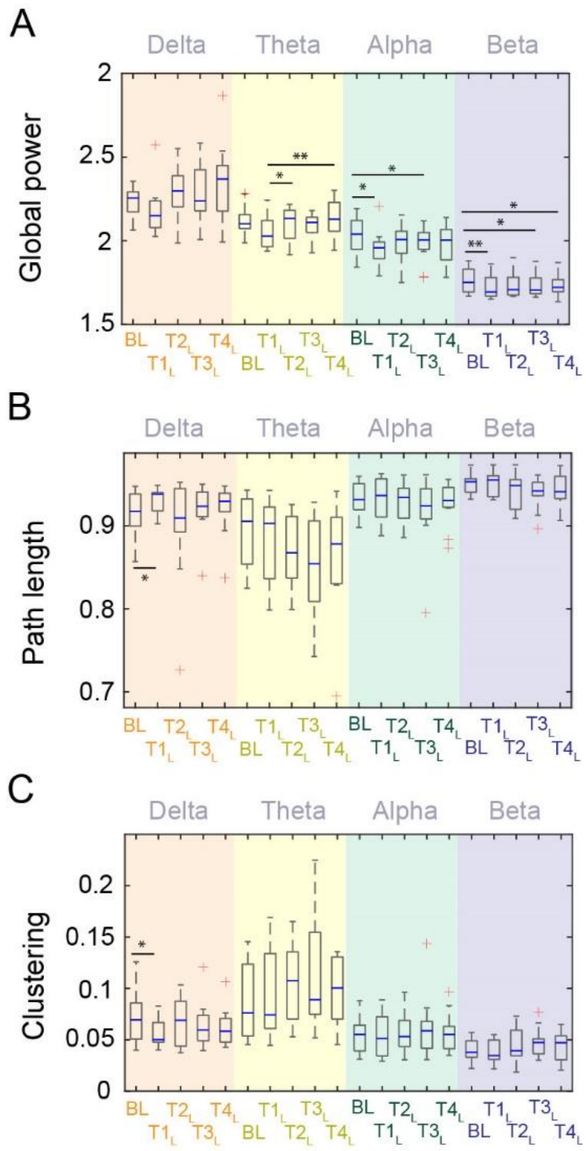

Figure S3: (A) Comparison for power, and (B) - (C) brain network properties across test bouts for each frequency band in the light intervention condition (blue-enriched light). BL = baseline, $T\#_L$ = Test bout # during the light condition. Asterisks represent significant difference on a paired *t*-test without any further correction applied such that **p* < .05 and ***p* < .01.

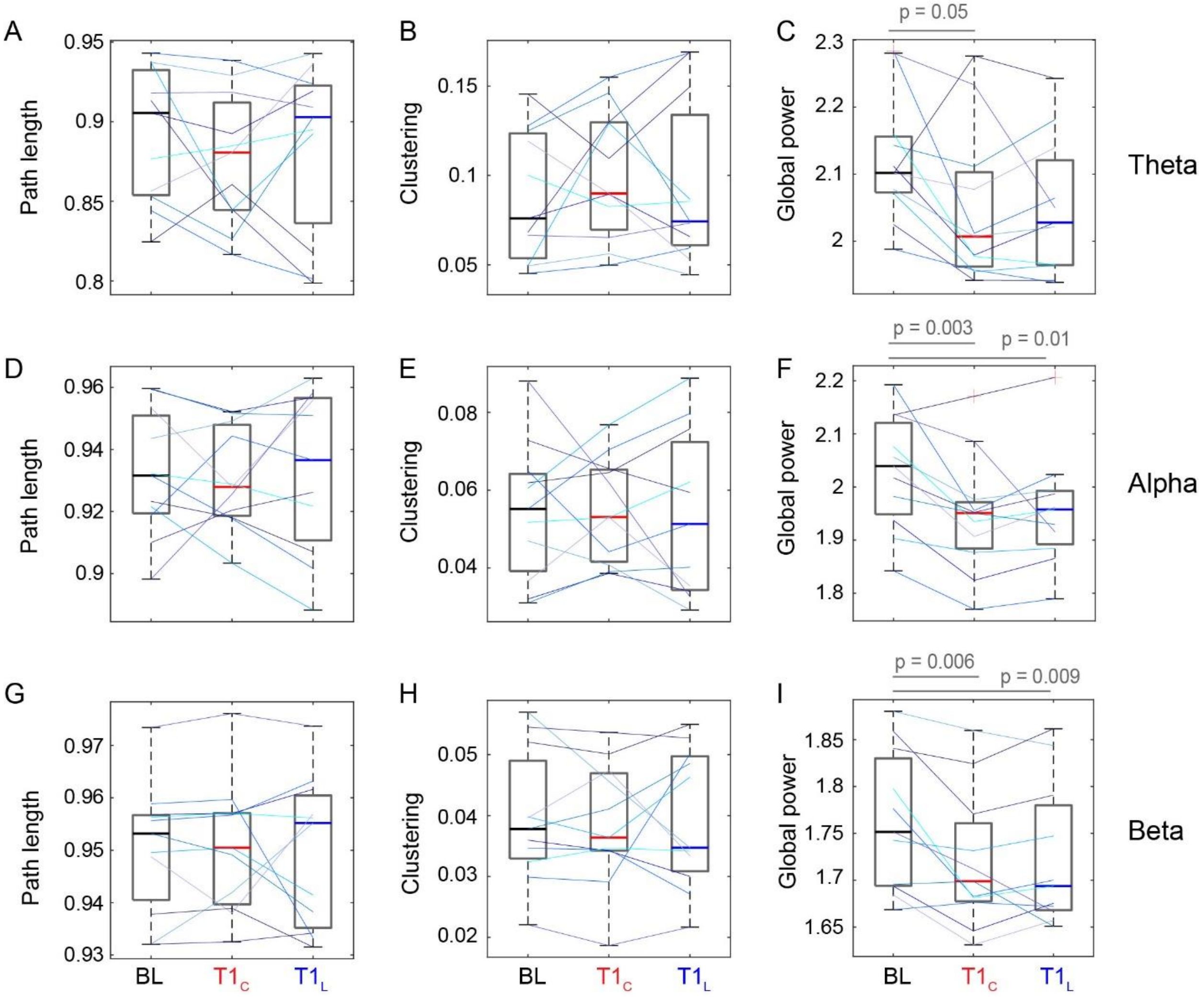

Figure S4: (A) - (B) Brain network properties comparing pre-sleep baseline (BL), control at T1 ($T1_C$), and light at T1 ($T1_L$) for different frequency bands; (C) Similar comparison for power. Colored lines represent individual participants.

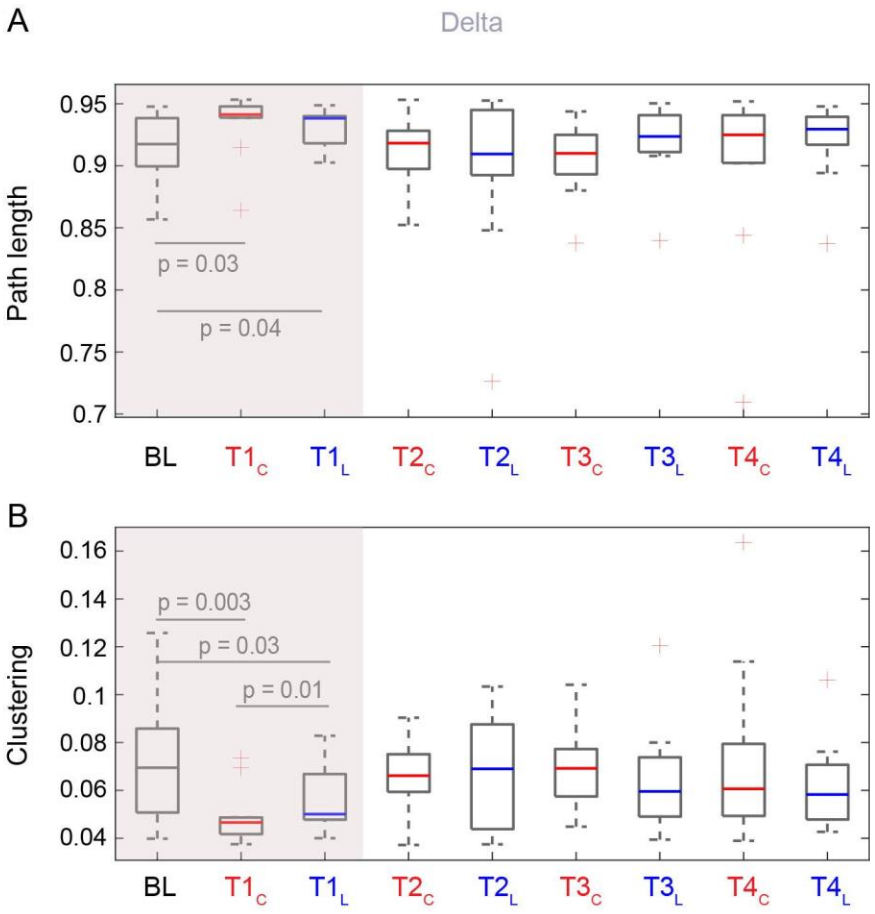

Figure S5: Brain network properties comparing pre-sleep baseline (BL), control (T#$_C$), and light (T#$_L$) at different test bouts (T1 to T4) for the delta frequency band. The shaded area is reproduced from Figure 3. Here, significant (< .05) *p*-values are indicated only for the *t*-test comparisons with the baseline and between control and light within each test bout.

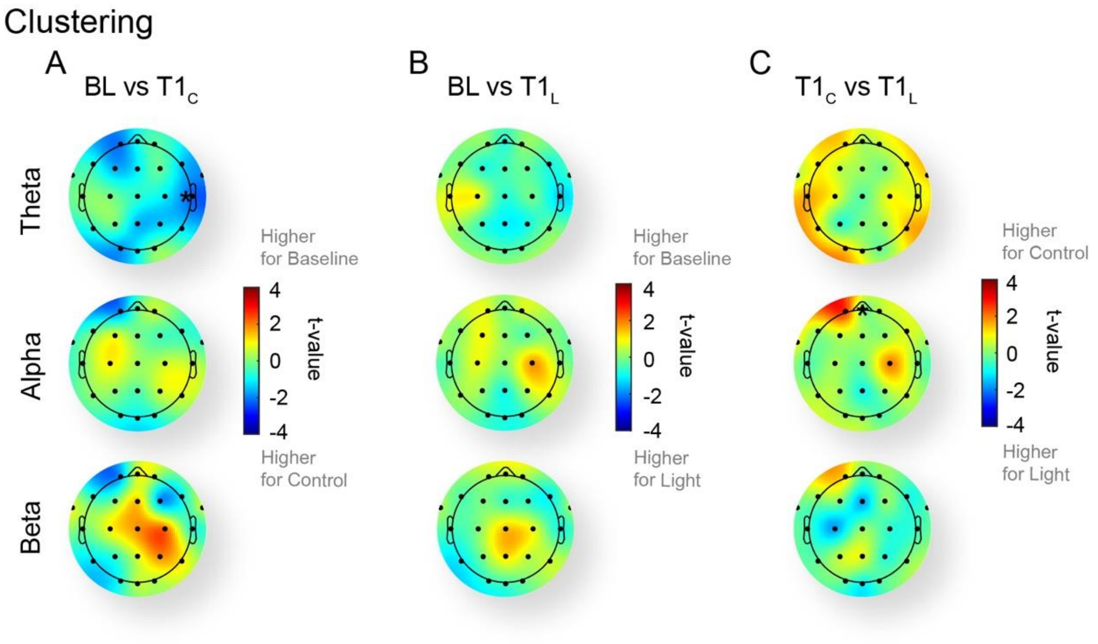

Figure S6: Change in clustering between (A) baseline (BL) and control at T1 ($T1_C$), (B) baseline and light at T1 ($T1_L$), and (C) control and light at T1 across scalp regions in the theta, alpha, and beta bands (complementing Figure 3 in the manuscript). Asterisks represent electrodes with significant difference on a paired *t*-test ($p < .05$). Electrodes that survived an additional correction for multiple comparisons are highlighted in white ($q < .05$).

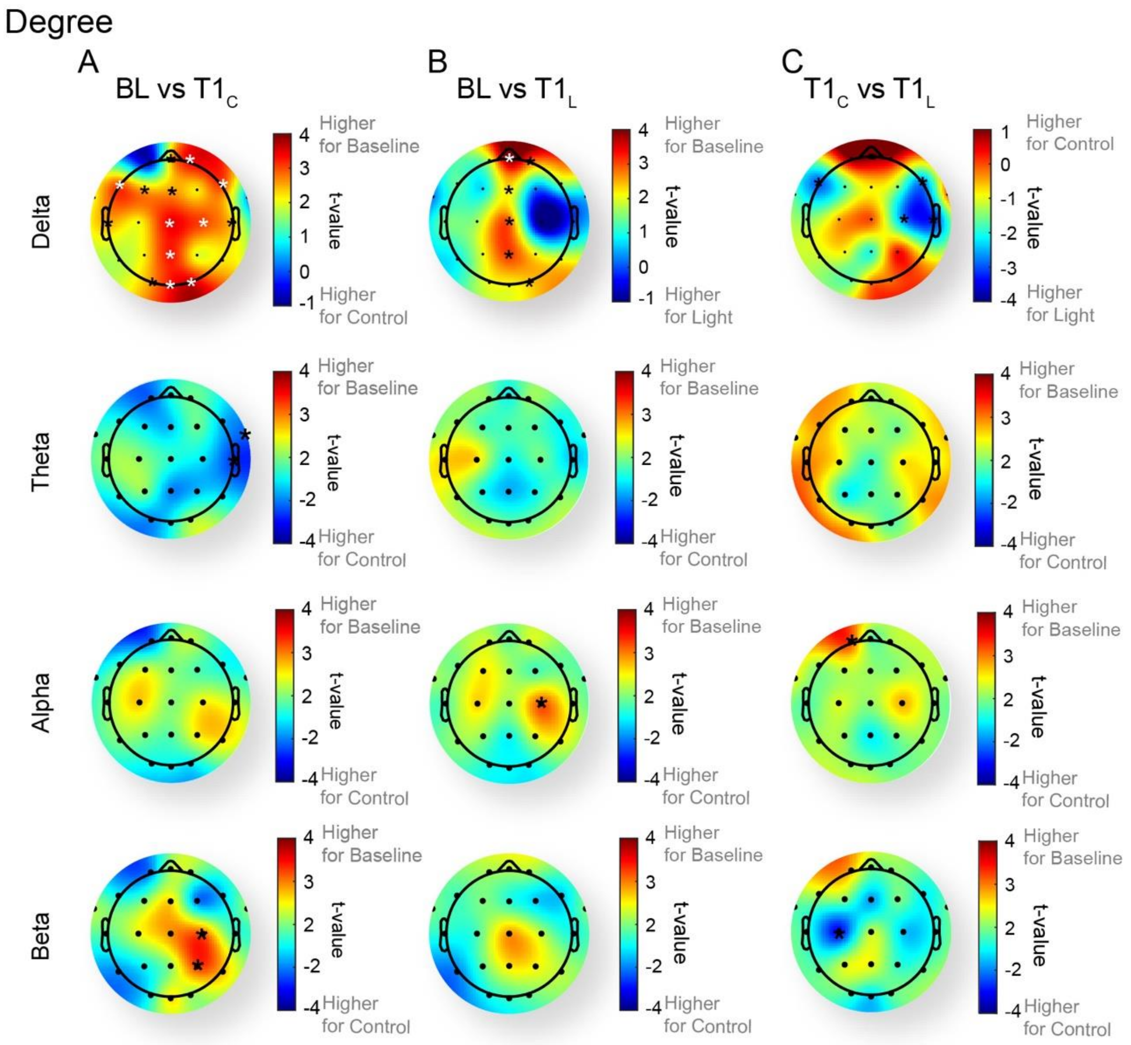

Figure S7: Change in degree between (A) baseline (BL) and control at T1 ($T1_C$), (B) baseline and light at T1 ($T1_L$), and (C) control and light at T1 across scalp regions in the theta, alpha, and beta bands (complementing Figure 3 in the manuscript). Asterisks represent electrodes with significant difference on a paired *t*-test ($p < .05$). Electrodes that survived an additional correction for multiple comparisons are highlighted in white ($q < .05$).

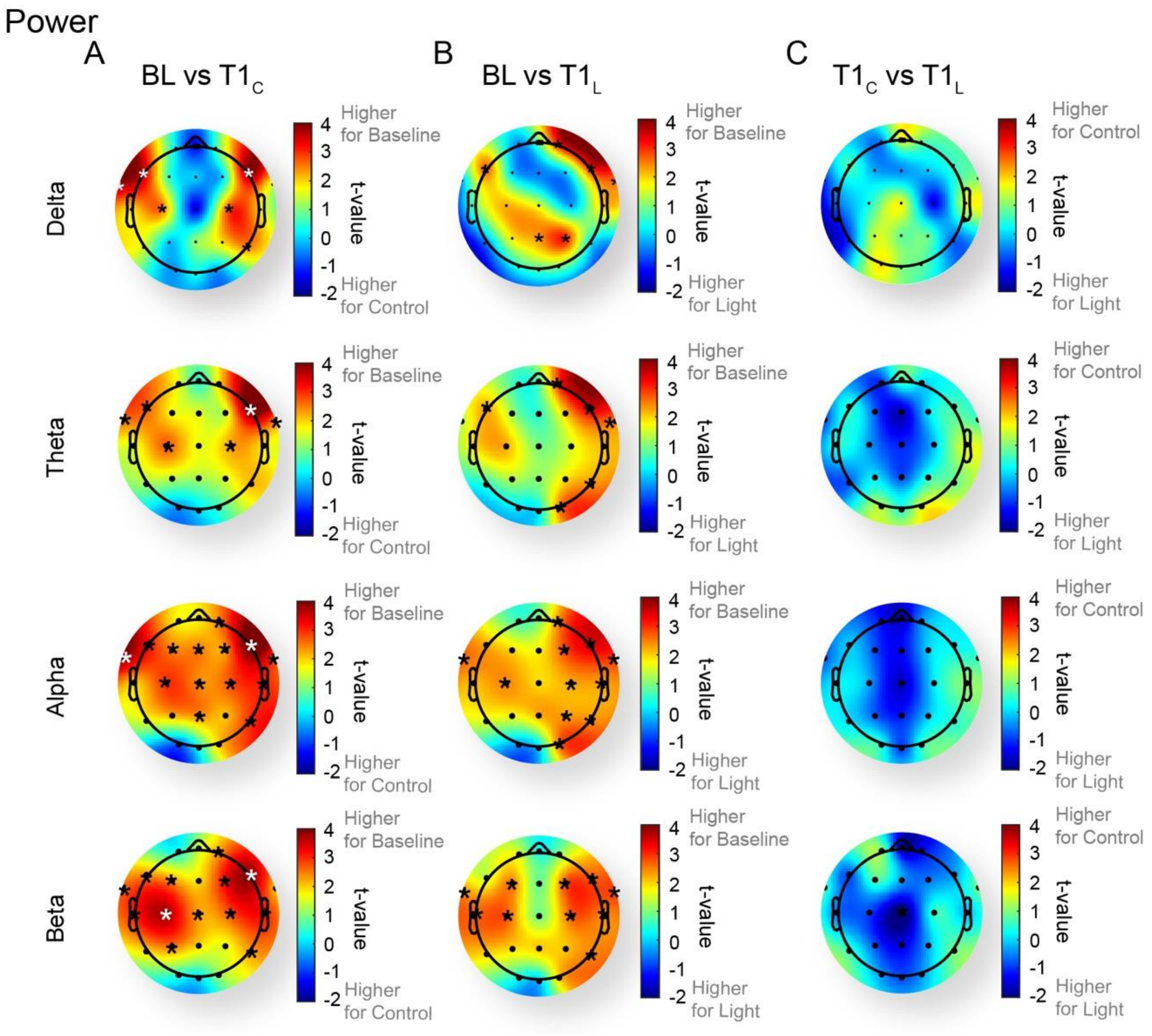

Figure S8: Change in spectral power between (A) baseline (BL) and control at T1 ($T1_C$), (B) baseline and light at T1 ($T1_L$), and (C) control and light at T1 across scalp regions in the theta, alpha, and beta bands (complementing Figure 3 in the manuscript). Asterisks represent electrodes with significant difference on a paired *t*-test ($p < .05$). Electrodes that survived an additional correction for multiple comparisons are highlighted in white ($q < .05$).

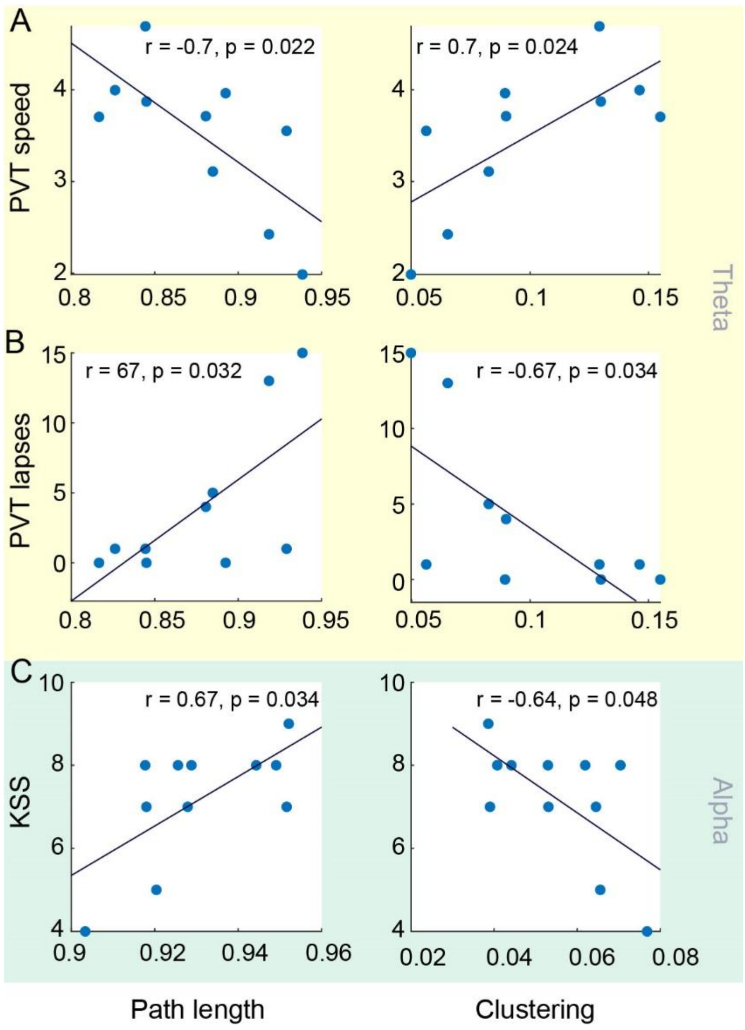

Figure S9: Relationship between neural and behavioral metrics. KSS = Karolinska Sleepiness Scale; PVT = psychomotor vigilance task. *r* and *p* represent Pearson's correlation coefficient and the associated *p*-value respectively.

Supplemental References

Donoghue T, Haller M, Peterson EJ, Varma P, Sebastian P, Gao R, Noto T, Lara AH, Wallis JD, Knight RT, Shestyuk A, & Voytek B (2020). Parameterizing neural power spectra into periodic and aperiodic components. *Nature Neuroscience*, 23, 1655-1665. DOI: 10.1038/s41593-020-00744-x

Leske S, & Dalal SS (2019). Reducing power line noise in EEG and MEG data via spectrum interpolation. *Neuroimage*, 189, 763-776. doi: 10.1016/j.neuroimage.2019.01.026

Oostenveld R, Fries P, Maris E, & Schoffelen JM (2011). FieldTrip: open source software for advanced analysis of MEG, EEG, and invasive electrophysiological data. *Computational Intelligence and Neuroscience*, 2011, 156869. https://doi.org/10.1155/2011/156869
